# Hunting promotes spatial reorganization and sexually selected infanticide

**DOI:** 10.1038/srep45222

**Published:** 2017-03-23

**Authors:** M. Leclerc, S. C. Frank, A. Zedrosser, J. E. Swenson, F. Pelletier

**Affiliations:** 1 Canada Research Chair in Evolutionary Demography and Conservation & Centre for Northern Studies, Département de biologie, Université de Sherbrooke, Sherbrooke, J1K2R1, Canada; 2 Faculty of Technology, Natural Sciences, and Maritime Sciences, Department of Natural Sciences and Environmental Health, University College of Southeast Norway, N-3800 Bø i Telemark, Norway; 3 Department of Integrative Biology, Institute of Wildlife Biology and Game Management, University of Natural Resources and Life Sciences, Vienna, Gregor Mendel Str. 33, A - 1180 Vienna, Austria; 4 Faculty of Environmental Sciences and Natural Resource Management, Norwegian University of Life Sciences, PO Box 5003, NO - 1432 Ås, Norway; 5 Norwegian Institute for Nature Research, NO-7485 Trondheim, Norway

## Abstract

Harvest can affect the ecology and evolution of wild species. The removal of key individuals, such as matriarchs or dominant males, can disrupt social structure and exacerbate the impact of hunting on population growth. We do not know, however, how and when the spatiotemporal reorganization takes place after removal and if such changes can be the mechanism that explain a decrease in population growth. Detailed behavioral information from individually monitored brown bears, in a population where hunting increases sexually selected infanticide, revealed that adult males increased their use of home ranges of hunter-killed neighbors in the second year after their death. Use of a hunter-killed male’s home range was influenced by the survivor’s as well as the hunter-killed male’s age, population density, and hunting intensity. Our results emphasize that hunting can have long-term indirect effects which can affect population viability.

Human activities are a major evolutionary force affecting wild populations^1^. There is increasing evidence that human exploitation leads to changes in morphological and life history traits worldwide^1^,^2^,^3^,^4^. For example, recent studies have shown that size-selective harvest by commercial fisheries and trophy hunting can induce evolution of heritable traits^5^,^6^,^7^,^8^,^9^. Harvest-induced evolution might not be desirable as the selection induced by human exploitation can be in the opposite direction of natural selection^10^,^11^,^12^.

Hunting can also have indirect effects on wildlife, although such effects are often ignored by managers, even though the removal of key individuals by hunting could change a population’s social structure^13^. For example, simulations suggest that the social networks of killer whales (*Orcinus orca*) may be vulnerable to targeted removal of individuals^14^. In African elephants (*Loxodonta africana*) the enhanced discriminatory abilities of the oldest individuals influences the social knowledge and reproductive success of entire groups^15^, suggesting that the loss of older individuals could decrease the fitness of all females within the group. In social species, the removal of any individual could affect social dynamics by changing the social structure. However, empirical evidence linking hunting and spatiotemporal reorganization of the social structure is lacking and the data needed to investigate this question are rarely available. Given the large number of species targeted by harvest, understanding the potential effects of removal on subsequent space use, social structure, and the fitness consequences for surviving individuals is critical to achieve sustainable hunting practices.

Here, we used detailed individual behavioral information from a Scandinavian brown bear (*Ursus arctos*) population (monitored from 2008–2015) to evaluate whether surviving adult males (hereafter referred to as survivors) shift their home range use after a neighboring adult male has been killed by hunting (Table S1). We further investigated the intrinsic and extrinsic factors driving the spatiotemporal reorganization of male spatial structure. In this population, the removal of adult males through hunting increases the risk of sexually selected infanticide (SSI)^16^,^17^, which is a major determinant of population growth^18^. Although important for sustainable wildlife management^19^, the mechanism behind the harvest-induced increase of SSI remains unknown [but see Loveridge *et al*.^20^]. Spatial reorganization due to hunting of males may be the responsible mechanism, by increasing the probability that a female will encounter a new male that is unlikely to be the father of her cubs^13^,^16^.

## Results

**Figure 1:**
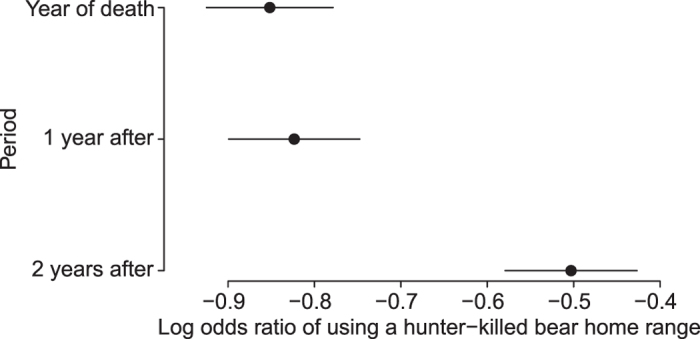
Changes in surviving male brown bears use of hunter-killed neighboring males’ home ranges over time. Shown are the coefficients and 95% confidence intervals for three consecutive years, i.e. the year the hunter-killed male was shot (baseline) and the following two years.

We found that survivors increased their use of the home ranges of hunter-killed males in the second year after their death (Fig. 1, Table S2). This time lag in the response likely is related to the bear’s ecology. Bears den from October to April^21^,^22^, shortly after the hunting season in late August—September. The size of the annual home range in our study population is mainly defined by space use during the mating season (May to mid-July), when males exhibit a roam-to-mate behavior^23^. Therefore, we hypothesize that survivors do not readjust their home range until after the first mating season without the hunter-killed neighbor. This could explain the two-year time lag in spatial reorganization. Our results support the contention that the spatiotemporal reorganization of male home ranges is an important mechanism linking hunter harvest to an increase in SSI, described above. It is also consistent with earlier studies in the same population showing lower cub survival following a two-year time lag after a male had been killed^16^,^17^.

**Figure 2:**
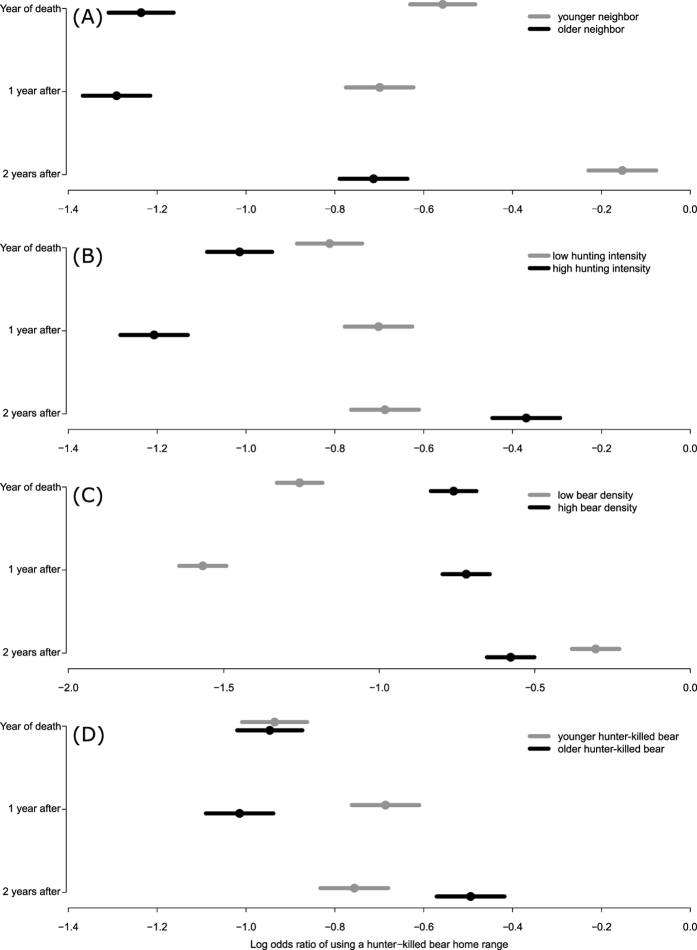
Influence of intrinsic and extrinsic factors on the speed and strength at which a surviving male will use hunter-killed neighboring males’ home ranges. Shown are the coefficients and 95% confidence intervals for three consecutive years, i.e. the year the hunter-killed male was shot (baseline) and the following two years, depending on the surviving male’s age (**A**), hunting intensity (**B**), population density (**C**), and hunter-killed male’s age (**D**), The low and high values in each panel represent the 25^th^ and 75^th^ percentiles, respectively, observed in the database.

We further investigated which intrinsic (ages of hunter-killed and surviving males) and extrinsic factors (population density and hunting intensity) modulated the speed and strength of the survivors’ response to hunting removals (Fig. 2, Tables S3 and S4). The use of a hunter-killed male’s home range by its surviving neighbors was influenced by (in order of decreasing relative importance) survivor’s age (∆BIC = 115), hunting intensity (∆BIC = 76), population density (∆BIC = 74), and hunter-killed male’s age (∆BIC = 6). Older survivors used a hunter-killed male’s home range less strongly following the hunter-killed male’s death than younger survivors (Fig. 2A). This suggests that older males may already have held home ranges with better resources, including food and females. Age-dependent home range quality could also explain why survivors increased their use of an old hunter-killed male’s home range more than that of a younger hunter-killed male (Fig. 2D).

Survivors more strongly increased their use of a hunter-killed male’s home range in the second year after its death when hunting intensity was greater (Fig. 2B). As increasing hunting intensity will increase the number of openings for surviving males, this should lead to a higher degree of spatial reorganization. We previously reported that the killing of an adult male within 25 km of a female strongly reduced the survival of her cubs, with a two-year time lag, although an increase in the number of killed males within 25 km had no significant additive effect^17^. Even though the degree of spatial reorganization increased with increased hunting intensity, this might not always translate into a correspondingly lower cub survival, because even though more surviving males may respond to increased hunting removal, only one infanticidal male is sufficient to kill most of females’ cubs. The other extrinsic factor affecting shifts in a survivor’s home range use was population density (Fig. 2C). Survivors at higher densities had higher initial overlap with the hunter-killed male and showed a weaker reorganization response than survivors at lower densities (Fig. 2C). Stronger competition for space between neighbors might explain why we observed higher initial overlap, with a weaker response at higher densities.

## Discussion

We identified a key behavioral mechanism linking hunting to an increase in SSI and show how post-hunt spatiotemporal reorganization of males was modulated by both intrinsic and extrinsic factors. By removing males from the population, hunters destabilized the spatial organization of the population for at least two years after a male had been killed. This period of two years might be specific to brown bears, due to their denning period and could be different in other harvested species with SSI, such as lions (*Panthera leo*)^20^ or cougars (*Puma concolor*)^24^. Nevertheless, hunting increases shifts in home range use by surviving males and increases the probability of SSI^16^,^17^. Male bears seem to assess their paternity through their mating history^25^, and increasing the magnitude of shifts in home range use would increase the probability that a male could encounter a female with whom he had not previously mated. Such a pattern is expected regardless of the cause of death (e.g., vehicle collision, management kill, natural mortality). However, hunting is often additive to natural mortality, as in our study system^26^, which increases the occurrence of SSI compared to unharvested systems.

The spatial distribution of the hunting mortality of bears was not homogenous in our study area^27^. Spatial and social relationships of bears are likely to change more rapidly in areas with higher hunting mortality, thereby potentially decreasing the cohesion of their social network^28^,^29^ but see ref. 30. Such effects could also influence the female reproductive rate because female brown bears exhibit kin-related spatial structures^31^, where neighbors negatively affect each other’s probability of having cubs^32^,^33^. The direct effect of removals due to hunting, in addition to the indirect effects of increasing cub mortality due to SSI and the potential impacts of decreasing social network cohesion, all increases heterogeneity in survival and reproductive rates. These effects combined could increase demographic variability and ultimately affect effective population size^34^,^35^. Therefore, we expect spatially structured demographic variability that could potentially result in source-sink dynamics^35^,^36^.

Our study sheds light on the importance of animal behavior to explain time lags in the responses to hunting in the wild. Understanding the indirect consequence of hunting over long time scales is critical for developing sustainable management practices and for the viability of harvested populations.

## Methods

The study area was in south-central Sweden (61°N, 15°E) and was composed of bogs, lakes, and intensively managed coniferous forest stands. The dominant tree species were Norway spruce (*Picea abies*), Scots pine (*Pinus sylvestris*), lodgepole pine (*Pinus contorta*), and birch (*Betula spp*.). Elevations ranged between 150 and 725 m asl. Gravel roads (0.7 km/km^2^) were more abundant than paved roads (0.14 km/km^2^). See Martin *et al*.^37^ for further information about the study area.

We captured brown bears from a helicopter using a remote drug delivery system (Dan-Inject^®^, Børkop, Denmark). We determined sex at capture and extracted a tooth from unknown individuals for age determination^38^. We equipped bears with GPS collars (GPS Plus; Vectronic Aerospace GmbH, Berlin, Germany) programed to relocate a bear with varying schedules (≤1 hour intervals). See Fahlman *et al*.^39^ for details on capture and handling. All captured bears were part of the Scandinavian Brown Bear Research Project and all experiments, captures and handling were performed in accordance with relevant guidelines and regulations and were approved by the appropriate authority and ethical committee (Naturvårdsverket and Djuretiska nämden i Uppsala, Sweden).

### Spatial analysis

We used adult male bears ≥4 years in the analysis to exclude natal dispersers^40^. We did not include natal dispersers because all male dispersers moved outside the study area where too few or no other males were GPS-collared. In addition, females actively defend their cubs during infanticide attempts. Therefore, younger dispersing males that have not yet attain full body size are less likely to successfully commit SSI than older, larger and better established males^41^. We screened the relocation data of adult males and removed GPS fixes with dilution of precision values >10 to increase spatial accuracy. To reduce autocorrelation, we used a 6-hour minimum interval between successive positions for a given bear. We excluded bears in years for which an individual had <75% of days with GPS locations from 1 May to 30 September.

We used an approach adapted from resource selection functions [RSFs;^42^] developed by Bischof *et al*.^43^. For each GPS-collared hunter-killed male we (1) determined its annual 95% kernel home range for the active period (1 May to 30 September or the day before he was killed) of the year in which he was killed and (2) calculated a 40-km radius circular buffer centered on its home range centroid. This radius was used because it represents the distance within which 95% of home range centroids of successful mates occurred^44^ and the distance at which the effect of male removal on cub survival seems to disappear^17^. In a given year, we used GPS relocations of the hunter-killed male and all the GPS locations of surviving adult males within the buffer (hereafter called survivors) to (3) calculate a 95% kernel isocline (hereafter called sampling space). For each survivor, we (4) generated as many random than GPS relocations within the sampling space and (5) determined if GPS and random relocations were inside or outside the hunter-killed bear’s home range. We repeated steps 3–5 for 3 consecutive years, i.e. the year a hunter-killed male had been killed and the two following years. We updated the sampling space annually by keeping the hunter-killed males’ relocations the year he was killed constant for the three years, and used the appropriate relocations of survivors for each year. We only used survivors that were alive and monitored during the three-year period. We repeated these steps for each hunter-killed male. This enabled us to test whether survivors increased their use of a hunter-killed male’s home range the years following its death.

For each hunter-killed male we also extracted a population density index derived from county-level scat collections in Sweden. We used the method of Jerina *et al*.^45^ and summed the weighted values of an individual bear’s multiple scats across a grid of 10 × 10 km. This was carried out for each county separately, after which the distribution was corrected temporally, using county-level trends of the Large Carnivore Observation Index^46^,^47^, provided by the Swedish Association for Hunting and Wildlife Management. Lastly, we calculated a proxy of hunting intensity based on the number of dead adult males located within the 40-km radius circular buffer centered on a given hunter-killed male’s home range centroid over a 3-year period prior to its death [see Gosselin *et al*.^17^ for further details].

### Statistical analysis

As a first step, we determined if surviving males shifted their home range use in response to the removal of a hunter-killed male. To do so, we used a generalized linear mixed model (GLMM) with binomial distributed errors. We coded the dependent variable either as GPS (coded 1) or random (coded 0) relocation. As independent variables we used a dummy variable representing whether the relocations were inside (coded 1) or outside (coded 0) the hunter-killed males home range, as well as a variable representing the period of the relocations (3-level factor; the year of the hunter-killed male’s death, as well as 1 and 2 years after the hunter-killed male’s death). We evaluated 4 candidate models (Table S1) and selected the most parsimonious based on the Bayesian information criterion (BIC)^48^. To control for the effect of year and unequal sample sizes across individuals, we included Year and the survivor ID nested within the hunter-killed males’ ID as random intercepts in all candidate models.

In a second step, we examined how intrinsic (i.e., age of survivor and hunter-killed males) and extrinsic (i.e., population density and hunting intensity) factors influenced the speed and strength at which a survivor would adjust its home range use in response to the removal of a hunter-killed male. We used a GLMM with binomial distributed errors and coded the dependent variable either as GPS (coded 1) or random (coded 0) relocation. We evaluated the effect of six independent variables; inside vs outside the hunter-killed male home range, period, age of the survivor, age of the hunter-killed male, population density, and hunting intensity to build 17 candidate models (Table S3). We selected the most parsimonious model based on BIC. To control for the effect of year and unequal sample sizes across individuals, we included Year and the survivor ID nested within the hunter-killed males’ ID as random intercepts in all candidate models. To facilitate model convergence, we scaled (mean = 0, variance = 1) all numerical covariates. We assessed the relative importance of variables within the most parsimonious model by dropping each variable and monitoring the ∆BIC. The larger the relative difference in BIC compared to the most parsimonious model, the more important we considered a variable. For all candidate models tested, the variance inflation factor (VIF) value was <2^49^. We used R version 3.2.3 for all statistical analyses^50^.

We captured and GPS-monitored a total of 15 adult males between 2008 and 2015. The database contained 19,133 GPS and 19,133 random relocations of 11 hunter-killed males and 7 survivors, for a total of 23 survivor – hunter-killed male pairs.

## Additional Information

**How to cite this article**: Leclerc, M. *et al*. Hunting promotes spatial reorganization and sexually selected infanticide. *Sci. Rep.* **7**, 45222; doi: 10.1038/srep45222 (2017).

**Publisher's note:** Springer Nature remains neutral with regard to jurisdictional claims in published maps and institutional affiliations.

## Supplementary Material

Supplementary Information

Dataset 1
