# Supplementary material for: Hunting promotes spatial reorganization and sexually selected infanticide
Source: Sci Rep. 2017 Mar 23;7:45222. doi: 10.1038/srep45222 (PMC5362984; doi:10.1038/srep45222)
Supplement: Supplementary Information [file srep45222-s1.pdf]

Supplementary Information

Hunting promotes spatial reorganization and sexually selected infanticide

M. Leclerc, S.C. Frank, A. Zedrosser, J.E. Swenson, F. Pelletier

correspondence to: Martin.Leclerc2@USherbrooke.ca

Table S1. Candidate models tested to determine if surviving adult male brown bears shifted their home range use in response to the removal of a neighboring hunter-killed male. Candidate models included different combinations of 2 variables: if the relocation was inside or outside the hunter-killed male's home range (Inside) and if the relocations were 0, 1, or 2 years after the hunter-killed male's death (Period). All candidate models also included Year and survivor ID nested in the hunter-killed males' ID as random intercepts. Models are listed with their number of parameters (K), Log Likelihood (LL), difference in BIC relative to the most parsimonious model (ΔBIC), and model weight (ω).

Model	Variable	K	LL	ΔBIC	ω
1	None	4	-26524	893.6	0.00
2	Inside	5	-26077	9.6	0.01
3	Period	6	-26524	914.7	0.00
4	Inside × Period	9	-26051	0.0	0.99

Table S2. Coefficients and 95% confidence intervals of the variables included in the most parsimonious model tested to determine if surviving adult male brown bears shifted their home range use in response to the removal of a hunter-killed adult male. See Table S1 for variable descriptions.

Variable	β	95 % Confidence intervals	
		Lower	Upper
Intercept	0.265	0.180	0.350
Inside = True	-0.852	-0.928	-0.775
Period = 1 year after male death	-0.001	-0.060	0.058
Period = 2 years after male death	-0.081	-0.142	-0.019
Inside = True × Period = 1 year after male death	0.028	-0.077	0.134
Inside = True × Period = 2 years after male death	0.349	0.242	0.455

Table S3. Candidate models tested to determine how intrinsic and extrinsic factors influenced shifts in home range use by surviving adult male brown bears in response to the removal of a neighboring hunter-killed adult male. Models include different combinations of 6 variables: if the relocation was inside or outside the hunter-killed male home range (Inside), if the relocations was 0, 1, or 2 years after the hunter-killed male's death (Period), the hunter-killed male's age (F_{age}), the surviving male's age (N_{age}), the harvest intensity (Harvest), and the population density index (Density). Models are listed with their number of parameters (K), Log Likelihood (LL), difference in BIC relative to the most parsimonious model (ΔBIC), and model weight (ω).

Model	Variable	K	LL	ΔBIC	ω
1	None	4	-26524	1206	0
2	Inside × Period	9	-26051	312	0
3	F_{age} × Inside × Period	15	-26044	361	0
4	N_{age} × Inside × Period	15	-25885	43	0
5	Harvest × Inside × Period	15	-25978	230	0
6	Density × Inside × Period	15	-25940	154	0
7*	F_{age} + N_{age}	21	-25865	67	0
8*	F_{age} + Harvest	21	-25961	260	0
9*	F_{age} + Density	21	-25921	180	0
10*	N_{age} + Harvest	21	-25858	53	0
11*	N_{age} + Density	21	-25847	31	0
12*	Harvest + Density	21	-25898	133	0
13*	F_{age} + N_{age} + Harvest	27	-25837	74	0
14*	F_{age} + N_{age} + Density	27	-25838	76	0
15*	F_{age} + Harvest + Density	27	-25857	115	0
16*	N_{age} + Harvest + Density	27	-25803	6	0.05
17*	F_{age} + N_{age} + Harvest + Density	33	-25768	0	0.95

* Each variable in models 7–17 is included with its interaction with the covariates "Inside" and "Period" as in models 3–6.

Table S4. Coefficients (β) and 95% confidence intervals of the variables included in the most parsimonious model tested to determined how intrinsic and extrinsic factors influenced shifts in home range use by surviving adult male brown bears in response to the removal of a neighboring hunter-killed adult male. See Table S3 caption for variable descriptions.

Variable	β	95 % Confidence intervals	
		Lower	Upper
Intercept	0.250	0.159	0.341
F_{age}	-0.117	-0.218	-0.017
N_{age}	0.027	-0.043	0.097
Harvest	-0.118	-0.216	-0.020
Density	-0.024	-0.129	0.081
Inside = True	-0.947	-1.037	-0.857
Period = 1 year[a]	0.014	-0.052	0.080
Period = 2 years[b]	-0.085	-0.164	-0.005
Inside = True × Period = 1 year	-0.092	-0.224	0.039
Inside = True × Period = 2 years	0.473	0.351	0.594
F_{age} × Inside = True	-0.013	-0.123	0.098
F_{age} × Period = 1 year	0.089	-0.010	0.188
F_{age} × Period = 2 years	-0.050	-0.145	0.044
N_{age} × Inside = True	-0.653	-0.846	-0.459
N_{age} × Period = 1 year	0.005	-0.082	0.092
N_{age} × Period = 2 years	0.057	-0.027	0.140
Harvest × Inside = True	-0.220	-0.341	-0.098
Harvest × Period = 1 year	0.109	0.015	0.204
Harvest × Period = 2 years	-0.145	-0.246	-0.044
Density × Inside = True	0.375	0.254	0.496
Density × Period = 1 year	-0.067	-0.163	0.030
Density × Period = 2 years	0.124	0.028	0.221
F_{age} × Inside = True × Period = 1 year	-0.378	-0.545	-0.211
F_{age} × Inside = True × Period = 2 years	0.325	0.165	0.485
N_{age} × Inside = True × Period = 1 year	0.083	-0.174	0.341
N_{age} × Inside = True × Period = 2 years	0.114	-0.115	0.343
Harvest × Inside = True × Period = 1 year	-0.328	-0.501	-0.155
Harvest × Inside = True × Period = 2 years	0.564	0.403	0.726
Density × Inside = True × Period = 1 year	0.267	0.087	0.446
Density × Inside = True × Period = 2 years	-0.582	-0.750	-0.415

[a] 1 year after the hunter-killed male death, [b] 2 years after the hunter-killed male death
